# Precluding rare outcomes by predicting their absence

**DOI:** 10.1371/journal.pone.0223239

**Published:** 2019-10-10

**Authors:** Eric W. Schoon, David Melamed, Ronald L. Breiger, Eunsung Yoon, Christopher Kleps

**Affiliations:** 1 Department of Sociology, The Ohio State University, Columbus, Ohio, United States of America; 2 School of Sociology, University of Arizona, Tucson, Arizona, United States of America; University of Edinburgh, UNITED KINGDOM

## Abstract

Forecasting extremely rare events is a pressing problem, but efforts to model such outcomes are often limited by the presence of multiple causes within classes of events, insufficient observations of the outcome to assess fit, and biased estimates due to insufficient observations of the outcome. We introduce a novel approach for analyzing rare event data that addresses these challenges by turning attention to the conditions under which rare outcomes do *not* occur. We detail how configurational methods can be used to identify conditions or sets of conditions that would preclude the occurrence of a rare outcome. Results from Monte Carlo experiments show that our approach can be used to systematically preclude up to 78.6% of observations, and application to ground-truth data coupled with a bootstrap inferential test illustrates how our approach can also yield novel substantive insights that are obscured by standard statistical analyses.

## Introduction

Revolutions, economic crashes, and nuclear disasters are examples of extremely rare events [1–3]. Forecasting such rare events is a pressing problem, and at least three issues have hindered efforts to model these outcomes. First, the specific causes identified for one instance of a rare outcome may not (and often do not) generalize to other instances [4]. Second, many such events are rare enough that there is insufficient data to appropriately assess the extent to which the observations fit a statistical distribution, creating (often unseen) problems of inference [5]. Third, in such instances where there are numerically few instances where *Y =* 1, model estimates will be biased toward the zeroes and underestimate the probability of a 1 in finite samples [6–7].

We introduce a novel approach to analyzing rare event data that addresses these challenges by turning attention to the conditions under which rare outcomes do *not* occur (i.e., the vast majority of observations). We treat rare events as causally asymmetric, such that the conditions leading to the occurrence of the rare event are distinct from the conditions associated with non-occurrence [8]. Leveraging this causal asymmetry, we identify conditions that systematically preclude the non-occurrence of a rare event. This allows us to identify substantive patterns *surrounding* rare events without assuming that the rare events themselves fit a uniform pattern or distribution. Thus, our method complements other important advances in the analysis of rare events such as Probabilistic Graphical models [9], Bayesian models [2, 10], integrated human/machine learning procedures [11], and methods of statistical correction for biased coefficients [6].

To identify conditions or sets of conditions that would preclude the occurrence of a rare outcome we use configurational methods of analysis. Configurational methods are set-theoretic and presume that logical conjunctions of conditions are causally relevant for producing an outcome [12–15]. These methods permit that a single condition (or combination of conditions) can predict both the presence and the absence of the outcome, depending on the other conditions with which it combines. However, configurational methods do not presume that solutions identified for the presence of a phenomenon are the arithmetic inverse of those identified for predicting its absence, and thus configurational methods facilitate the analysis of causally asymmetric phenomena [13, 16]. We identify conditions or configurations of conditions with 100% consistency with the absence of the rare outcome. This calculation is presented in Eq 1, where X represents a causal condition or set of causal conditions and Y represents the outcome [8]. The equation specifies the proportion of observations manifesting X that also manifest Y (i.e., the extent to which X represents a subset of Y). Here, inverting the analysis so that the *absence* of the rare outcome is coded “1”, if all X manifest the absence of the outcome, then the solution to Eq 1 must equal 1 and we speak of 100% consistency, thus ensuring that no observations of the rare outcome are associated with the X.

$$\left({\text{X}}_{i}\leq {\text{Y}}_{i}\right)=\frac{\sum {\text{min(X}}_{\text{i}}{\text{,Y}}_{\text{i}}\text{)}\;}{\sum {\text{X}}_{\text{i}}}$$ (1)

Using Monte Carlo (MC) experiments, we show how analyzing the absence of a rare outcome in this way can be used to reduce sample size. The experiment assesses case reduction sensitivity to variation in sample size and the rarity of the outcome (Y). If predicting extremely rare events is like finding a needle in a haystack, our approach effectively reduces the size of the haystack. Thus, we improve the chances of predicting rare outcomes by identifying conditions that might preclude the occurrence of a rare event. We also apply our approach to a published analysis of an extremely rare event: violent political infighting. In addition to demonstrating the utility of our approach for the purposes of reducing sample size, this re-analysis reveals findings that are novel and complementary to the conclusions published in the original analysis of this data.

## Methods and materials

### Technical background: Truth tables and boolean minimization

To analyze the absence of rare events, we begin by constructing a truth table. A truth table is a two-dimensional array of *k*+2 columns and *d* rows, where *k* represents the number of predictors/conditions and *d* is the number of logically possible configurations. The number of rows for a truth table is equal to 2^k^. The number of logically possible configurations therefore increases exponentially as predictors are added. For each row of the truth table, the consistency (Eq 1) between the configuration and the outcome is calculated based on the observed data that corresponds to the configuration represented.

An example of a truth table representing the four logically possible combinations of conditions associated with two predictors (X1, X2) is given in Table 1. N indicates the number of cases associated with each configuration. The Observed N of Outcome indicates the number of cases where the outcome of interest is present, and Assigned Outcome Value indicates whether the hypothesis that the observations associated with a configuration represent a subset of the outcome is considered to be true. As stated above, Consistency refers to the extent to which the cases in each configuration represent a subset of the outcome. When all data are coded as binary, the consistency is equivalent to the conditional probability of Y for observations exhibiting each configuration. The first two configurations are associated with the presence of Y, whereas the second two configurations are associated with the absence of Y.

**Table 1:** Truth table example.

X1	X2	N	Observed N of Outcome	Assigned Outcome Value	Consistency
1	0	2	2	1	1
0	1	5	4	1	0.8
1	1	3	2	0	0.67
0	0	4	2	0	0.5

The purpose of the truth table is to identify configurations that logically imply Y. It is often the case that analysts will choose some threshold for consistency below 1.00 (0.8 and 0.9 are commonly used as cut-points; *8*) as indicating that a configuration logically implies Y (i.e., Y = 1 in the truth table). This is based on the assumption that lower thresholds nevertheless imply a subset relationship. For illustrative purposes, our example truth table uses 0.8 as the cutpoint, coding Y = 1 for the two configurations where Consistency is at least 0.8. In Table 1, the configuration represented in the first row is found in two observations, and both observations also exhibit our outcome of interest. The second configuration is found in five observations, four of which also exhibit the outcome of interest. The above truth table example shows that configurations X1*~X2 = Y and ~X1*X2 = Y, but X1*X2 = y and ~X1*~X2 = y, where * implies a logical AND statement, the symbol ~ indicates the absence of a condition, and a lower case “y” indicates the absence of the outcome. Thus, the truth table indicates that X1 logically implies Y only when X2 is absent, and not when X2 is present.

For the purposes of our experimental analysis and our analysis of ground-truth data, we constructed truth tables of all logically possible configurations of conditions, then eliminated any configurations that were not exhibited in any observations in the dataset. Thus, even though 20 predictors (the number included in our experimental models) logically implies 1,048,574 possible configurations, the number of configurations accounted for was logically limited to the N of the data, though functionally the number of configurations was lower due to patterning among the data (i.e., multiple observations associated with a given configuration). For each of these configurations, we calculated the consistency of each solution with the outcome. Our goal is to identify configurations where the conditional probability of Y is equal to 1 (i.e., identify configurations where the probability of the absence of a rare event is 1.000). This allows us to ensure that no observations where Y = 0 are represented by those configurations. Because we are inverting our analysis such that the absence of a rare event is the outcome of interest, this allows us to identify configurations where the rare outcome never occurs in the data.

In our Monte Carlo experiments, we identified all configurations that were 100% consistent with the absence of the rare outcome and associated with a minimum of at least two observations in the data. We then calculated the coverage (Eq 2) to assess the extent to which removing the data associated with those configurations would allow us to reduce the total population of the data. Coverage is defined in Eq 2, where X represents a causal condition or set of causal conditions and Y represents the outcome. Coverage corresponds to the proportion of observations having Y = 1 that also manifest X. Because the goal of the experimental procedures was to assess the extent to which this method allows us to identify conditions under which a rare event will not occur, we sought only to identify what we considered to be minimal patterns (i.e., at least two cases) among the data.

$$\left({\text{Y}}_{i}\leq {\text{X}}_{i}\right)=\;\frac{\sum \text{min}{\;\text{(X}}_{\text{i}}{\text{,Y}}_{\text{i}}\text{)}}{\sum {\text{Y}}_{\text{i}}}$$ (2)

For our analysis of the ground-truth data, we included two additional dimensions in our analysis: 1) we used Boolean minimization to identify more parsimonious configurations, and 2) we applied stricter criteria for what we considered to represent an empirical pattern. As noted in the main text, the threshold for assuming that patterns among the data are empirically meaningful should be determined by the analyst for each analysis. Our application of stricter criteria was for purely illustrative purposes. For the analysis of ground-truth data, we selected 10% as our threshold, and only considered configurations with coverage scores of at least 0.10.

The goal of Boolean minimization is to eliminate as many conditions as possible from each set relation, and this is achieved by identifying conditions that are irrelevant because the outcome is unaffected by any of its values [15]. As above, * implies a logical AND statement. The symbol + implies a logical OR statement. Assume the following configuration is associated with the presence of Y:

X1*(~X2+X2)=(X1*~X2)+(X1*X2)

As Thiem and Dusa [15] write, by the laws of the excluded middle (one of the two distributive laws of Boolean algebra) where ~X2 + X2 = 1, it follows that X1*(~X2+X2) = Y, and therefore X1 = Y. Thus (X2 + ~X2) is redundant and only X1 is relevant with respect to Y. Moreover, X1 cannot be reduced further, and thus is a prime implicant representing the minimal union. Following this logic, configurations identified as consistent with the outcome in the truth table can be reduced to their minimal union, thus simplifying the expression of the logical configurations. From an applied perspective, this is a critical step as the complexity of configurations can obscure substantive insights.

For our ground-truth analysis, we use the Quine-McClusky algorithm [17–20] for minimizing the Boolean functions identified in our truth table analysis. Alternative algorithms exist (for a review and comparison of strengths and weaknesses, see ref. [15]), but this is by far the most common and was appropriate for our purposes and the complexity of our data. The Quine-McClusky algorithm begins by identifying all prime implicants of a function and uses those prime implicants to find the essential prime implicants of the Boolean function (for a complete elaboration, see ref. 18).

### Extensions to valued data

Configurational methods require data be constrained between 0 and 1. For ease of presentation, our experiments and ground-truth analysis use binary data. However, the approach also works with valued data, so long as they are constrained between 0 and 1. As detailed by Charles Ragin [8], measures can be calibrated to account for degrees of membership. There are basic differences in how set membership is assessed with fuzzy sets versus crisp sets, namely, there is not a 1:1 correspondence between truth tables for fuzzy sets versus truth tables when used with binary data. When analyzing binary data, all cases are defined as either in or out of the set defined by a given configuration. Thus, a single case can appear in only one row of the truth table. However, with fuzzy sets, each case can have partial membership in every logically possible configuration. As Ragin [21] writes, "When using a truth table to analyze the results of fuzzy-set assessments, the truth table rows do not represent subsets of cases, as they do in crisp-set analyses. Rather, they represent the 2^k^ causal arguments that can be constructed given a set of causal conditions." Consequently, whereas consistency in a crisp-set analysis is the conditional probability of the cases represented by a configuration exhibiting the outcome of interest, in a fuzzy set analysis the assessment of consistency for each configuration is based on *all* observations in the data, not just those with greater than 0.5 membership that row of the truth table. Nevertheless, our method can be applied to valued data so long as the threshold for consistency is maintained.

## Monte Carlo experiment

### Experimental design

The Monte Carlo experiment simulated a rare outcome and 20 predictor variables. All predictors were binary, and one of them was a “key predictor” that was associated with the outcome. For the key predictor, there was a 30% chance that it was coded ‘1’ when the outcome was a ‘1.’ The remainder of cases on the key predictor were coded ‘1’ with a probability equal to half the number of ones divided by the number of remaining of cases. The other 19 predictors were random draws from a binomial distribution with .159 probability of being a ‘1’ (i.e., > 1 on a standard unit normal distribution).

For each simulated dataset in our experiment, we identified combinations of conditions that met three criteria. First, the combinations of conditions must be associated with observed data. As noted above, with 20 predictors, there are 1,048,574 logically possible combinations of conditions. However, even with over a million logically possible combinations of conditions, if there are only 8,000 observations in the data there are a maximum of 8,000 possible combinations of conditions that are represented in the empirical data. Second, the observed data represented by each configuration must be 100% consistent with the absence of the rare outcome (Y = 0). A lower level of consistency indicates that the rare outcome that we are trying to rule out is empirically associated with that configuration. Third, the data associated with a combination must reflect a minimal degree of patterning among cases. For the purposes of our experiment, we defined a minimal degree of patterning as at least two observations being associated with a given configuration. While the first two criteria are invariant, the latter criteria can be adjusted upwards by the analyst to fit substantive or theoretical requirements.

For our experiments, we varied (*i*) the sample size (N = 500, 1,000, 2,000, 4,000 and 8,000), and (*ii*) the rarity of the outcome (frequency = 0.0025, 0.005, 0.01, 0.02, 0.04). We conducted this analysis 1,000 times for each of the different conditions in the Monte Carlo experiment. Thus, in our 5x5 experimental design, we estimated this procedure 25,000 times. Complete R code for replication is provided as supporting information in S1 Appendix.

## Results

**Fig 1:**
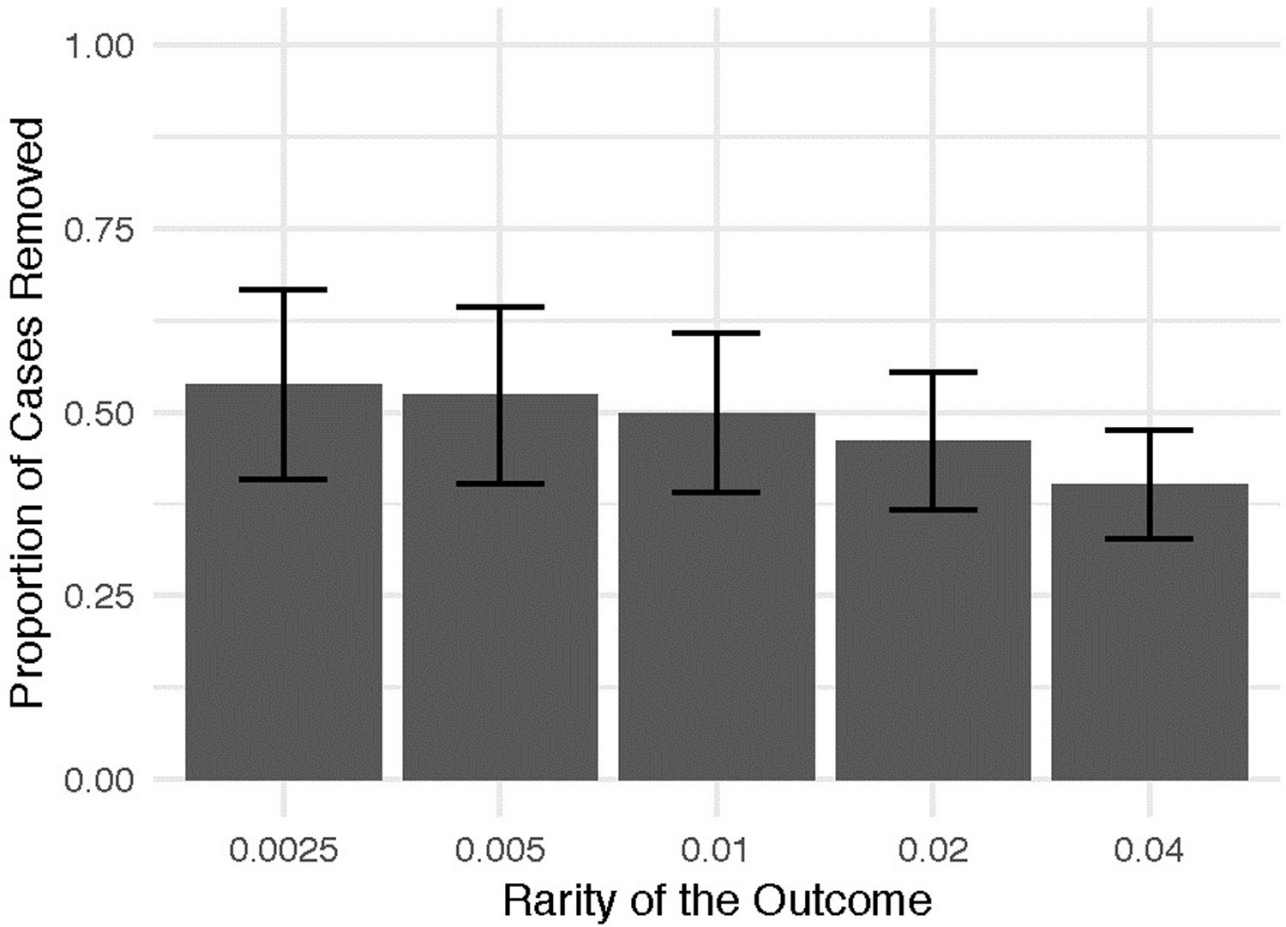
Proportion of cases removed by rarity of outcome. Error bars denote 95% confidence interval around the means.

Fig 1 presents the results of our experimental manipulation of the rarity of the outcome. Plots show average proportion of cases that were removed without removing any instance of the rare event and standard errors. In all analyses, we identified a non-trivial proportion of cases for removal, with a total range of 17.2% to 78.6%. Of interest is the proportion of cases identified for removal without risk of inadvertently removing cases that were coded “1” for the rare outcome. This proportion increased as the outcome became rarer. This finding is intuitive, given that the structure of the data that does not include the rare outcome becomes increasingly constrained as the number of cases exhibiting the rare outcome increases within the dataset. Nevertheless, even with 4% of the case exhibiting the rare outcome, the average proportion of cases removed was ~40%, and the observed minimum proportion of cases was non-zero.

**Fig 2:**
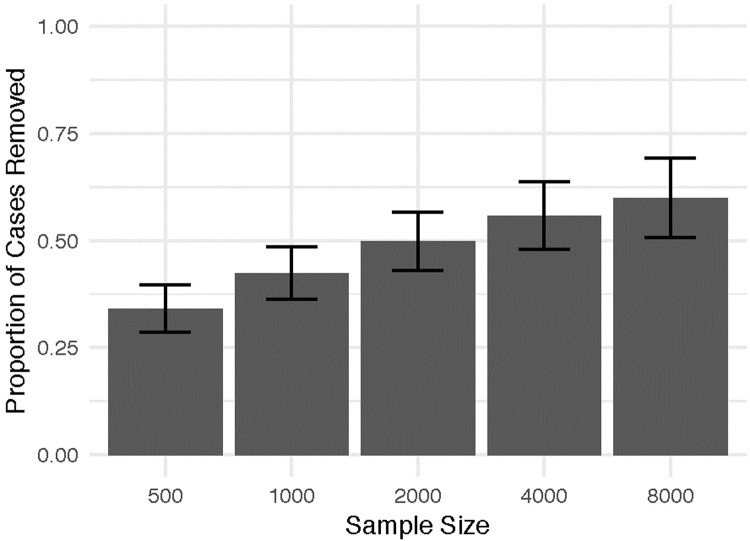
Proportion of cases removed by sample size. Error bars denote 95% confidence interval around the means.

Fig 2 shows the results of our experimental manipulation of sample size. Here, the patterning among cases—and thus, the proportion of cases that can be identified for removal—increases as the sample size increases. Notably, even with the smallest sample size, the proportion of cases removed represents a non-trivial reduction of cases, and the minimum proportion of cases removed remains non-zero.

Taken together, these results provide an illustration of using configurational methods to model the absence of a rare event for case removal. The experiment shows how a focus on identifying conditions that are associated with *non-occurrence* rather than the occurrence of a rare event allows us to identify patterns surrounding rare events. By focusing on the vast majority of cases (i.e., cases where the rare event does *not occur*)—which exhibit far more reliable patterns than those typically found among instances of the rare event—we circumvent the risk of assuming causal similarity between a small number of potentially unique cases (i.e., extremely rare events).

## Application to ground-truth data

### Ground-truth data

Our application to ground-truth data re-analyzed published data on violent political infighting, which was designed to replicate the statistical analyses presented in Wimmer, Cederman, and Min [22]. The unit of analysis for this replication data is country-years. Configurational analysis requires data constrained between 0 and 1 (either binary or valued data can be used, and both can be used in conjunction with the methods described here). For ease of presentation, we recoded the independent variables presented in ref. 16, Table 3, Model 1 as binary indicators. Using the published replication data we coded the data as follows:

- *Infighting* (outcome) - 1 if onsetstatus2 = 1, 0 if else
- *Excluded population—*0 if exclpop = 0, 1 if >0
- *Center segmentation—*0 if egipgrps ≤ 2; 1 if >2)
- *Imperial past—*0 if pimppast = 0, 1 if >0
- *Linguistic fractionalization*—0 if ethfrac ≤ 50, 1 if >50
- *High GDP* (measured per capita) - 0 if gdpcap ≤ 9.004, 1 if >9.005
- *Large population*—0 if popavg < 27000, 1 if ≥ 27000.1
- *Ongoing war—*0 if atwarnsl = 0, 1 if >0

In this list, the variable names as they appear in the published analysis are listed with the corresponding variable names as found in the published replication data.

The way that data are coded for the purposes of configurational analysis will influence the results of the analysis, and analysts should rely on substantive knowledge or theory to guide coding decisions as well as to interpret their findings [8, 21]. Because configurational methods are set-theoretic, a change in how a set is defined necessarily alters the criteria for set membership (i.e., if the definition of a large population changes from populations of 26 million to 27 million, then countries with 26.8 million will no longer be coded as “large”). For our analysis, we worked to identify logical sets by identifying substantively meaningful cut-points in the data. Our goal was not to develop a definitive set of categories, but rather to identify categories that drew substantively or theoretically salient distinctions, and would allow us to illustrate the utility of our methods. For example, Wimmer and colleagues construct a variable (*pimppast)* that measures the percentage of years that a country was under imperial rule from 1916 to independence [22]. Examining the data, we determined that a substantively meaningful division was between countries that had no imperial past versus countries that had any imperial past. We similarly distinguished between countries with no excluded population versus any excluded population to construct our measure of *Excluded population*.

In other cases, we relied on outside sources to inform our coding. For example, in coding our predictor *High GDP*, we used the World Bank’s income classifications of low income, lower middle income, upper middle income, and high income countries, which is based on a country’s Gross National Income (GNI). Wimmer and colleagues use a measure of GDP per capita standardized to US Dollars in the year 2000 as a proxy for levels of development. We calculated the range of GDP for each GNI category in the World Bank’s classification, then used those ranges to construct categories income categories consistent with the World Bank’s classification scheme. We included only a measure of high income in our analysis because Wimmer and colleagues specifically theorize that developed (i.e., higher income) countries are less likely to experience rebellions or infighting. Detailed explanations of the principles guiding our coding decisions for each predictor are available upon request.

Once the data were recoded, we dropped observations with missing values on any of the variables listed, resulting in a total dataset of 5,155 country-years of which 14 (0.27%) of the observations exhibited the rare outcome.

## Results

Table 2 presents the results of a logistic regression of violent political infighting using data published by Wimmer, Cederman, and Min [22]. To predict violent infighting, Wimmer and colleagues used multinomial logistic regression with the second outcome category being rebellion by political excluded. Because our interest is only in the rare event (violent infighting by politically included actors), we re-analyzed their data with a single binary outcome using logistic regression with standard errors clustered by country, thereby mimicking Wimmer and colleagues’ analysis in Table 3, Model 1, Column 1, except that we used a binary dependent variable rather than a multinomial outcome. The significance and direction of effects for our logistic regression correspond to those presented by Wimmer, et al [22], though coefficient estimates differ. Also, our logistic regression omits the coefficient for *Ongoing war* due to dependency. Wimmer and colleagues’ primary argument regarding infighting is that *Center segmentation*—defined as a political arrangement where power is shared between multiple ethnic elites who represent distinct interest groups—will increase the likelihood of infighting, and their statistical analysis supports this hypothesis. They also hypothesized that the likelihood of infighting will decrease as the *Excluded population* increases, and as states become larger. Their analysis supports the latter assertion, but not the former.

**Table 2:** Logistic regression of violent infighting.

Excluded population	-0.088
	(0.204)
Center segmentation	0.334**
	(0.117)
Imperial past	3.035
	(1.759)
Linguistic fractionalization	0.980
	(1.762)
GDP per capita	0.139
	(0.083)
Population size	-0.292*
	(0.143)
Ongoing war	(omitted)
	
Year	0.047**
	(0.016)
Peace years	0.080
	(0.358)
Spline 1	0.003
	(0.011)
Spline 2	-0.001
	(0.003)
Spline 3	0.000
	(0.001)
Constant	-99.135***
	(31.928)

*Note*: Standard errors are in parentheses.

*p< 0.05;

**p< 0.01;

***p< 0.001.

**Fig 3:**
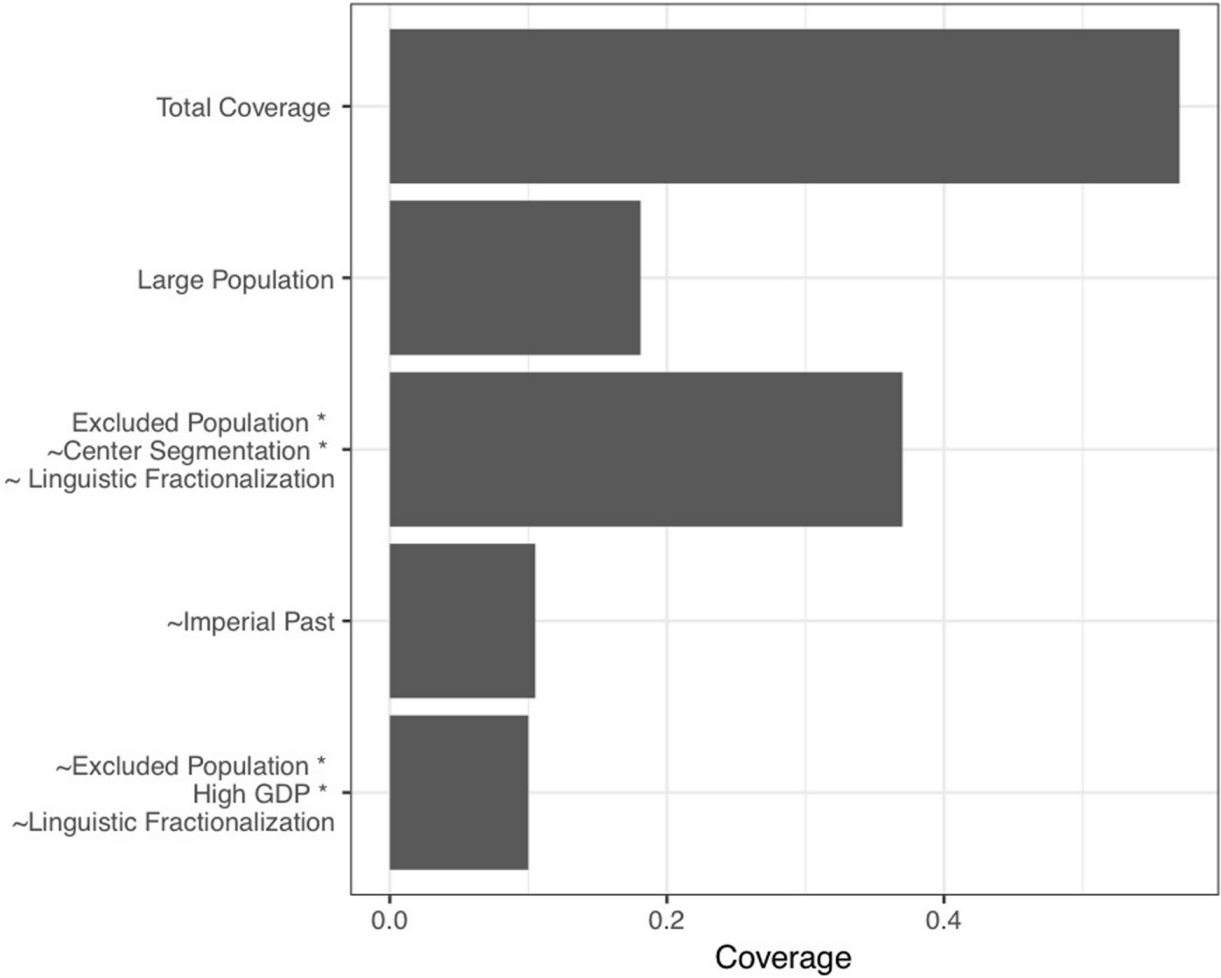
Configurational results for the absence of infighting. Bars denote proportion of cases removed overall, and for each configuration with a minimum of 10% coverage.

Fig 3 presents the results of configurational analysis for the *absence* of infighting after Boolean minimization and only including configurations where coverage is greater than or equal to 0.10. The Fig includes four conditions/configurations that are 100% consistent with the *absence* of infighting. In addition to listing the conditions/configurations that are entirely consistent with the absence of infighting, we also listed the solution coverage and the overall coverage. The complete truth table for our ground-truth analysis is provided as supporting information in S1 Table.

**Fig 4:**
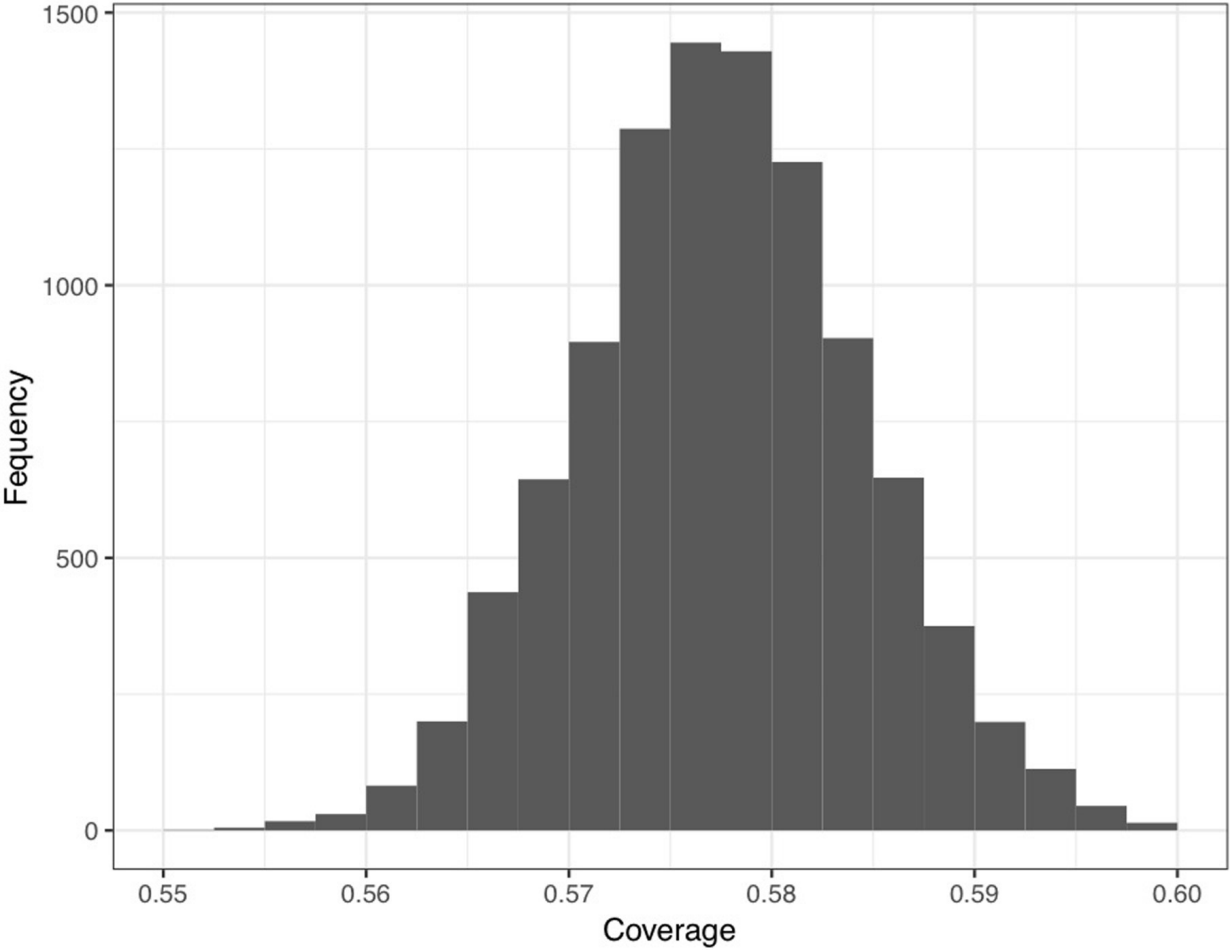
Distribution of coverage scores from bootstrap inferential test of the configurational analysis for the absence of infighting. Results indicate the coverage scores for the configurations presented in Fig 4 across 10,000 resamplings with replacement.

Together, the four configurations presented in Fig 3 allow us to reduce the population of data by 57%. Because the configurational analysis on its own provides only a descriptive analysis of patterns in the data, we used a bootstrap procedure to calculate a confidence interval around the descriptive point estimate [23]. We drew a random sample of 5,155 observations with replacement from the original dataset of 5,155 observations, then computed the descriptive conditional probability (coverage) that cases within the four configurations we identified were associated with the absence of infighting with 100% consistency. We repeated this procedure 10,000 times and calculated a 95% confidence interval. The 95% confidence interval was 0.5774–0.5776. Fig 4 shows a histogram of the distribution of coverage scores across the 10,000 resamplings.

Returning to the original data, we removed all observations that exhibit any of the four configurations identified in our analysis and re-ran the original logistic regression. Not only did the effect for *Center segmentation* remain, but model fit improved, as indicated by AIC and BIC statistics. Importantly, while model fit improved, the proportion of cases explained (as indicated by pseudo R-squared of the model) decreased in this particular analysis. The reason for this is that we removed cases that are well-explained by variables in this model. Consequently, the proportion of cases that are not explained well by this model now constitute a greater proportion of the data after the data reduction is completed. This indicates that an omitted variable may better explain the occurrence of the rare event, and thus an alternate model specification may be required to account for the unexplained variation in the model.

## Discussion

In conjunction, our Monte Carlo experiment and our re-analysis of Wimmer, Cederman and Min’s data illustrate the effectiveness of our method. By accounting for the causal asymmetry of rare events, we are able to leverage the fact that there are far more consistent patterns among the observations that do *not* exhibit the rare event than among observations that do. This allows us to circumvent analytically problematic discontinuities between instances of extremely rare outcomes while still identifying empirically meaningful patterns. By identifying patterns that are 100% consistent with the absence of the rare event, the method introduced here provides a systematic and empirically-driven way reduce a population of cases, which has been identified as a key element for improving model estimation and reducing bias in other methods for analyzing rare events. In particular, the results of our Monte Carlo experiment demonstrate the potential for non-trivial case reduction, thereby reducing the size of the metaphorical haystack. Our approach to case reduction thus allows analysts to identify relevant populations of observations where the occurrence of a rare event is empirically possible (i.e., to identify the population at risk for occurrence) while also identifying observations classes of observations where the rare event has never occurred. This is of particular utility for analyses of big data because it provides a powerful tool for addressing a key challenge of big data analysis—distinguishing between signal versus noise [24–25]—and future research should examine the potential for our approach to reduce computational time in very large datasets.

In addition to case reduction, shifting attention to identifying conditions or configurations of conditions that empirically preclude a rare event also yields substantively novel insights. This is illustrated by our re-analysis of Wimmer and colleagues’ data, which complements and extends those authors’ published findings. Our analysis identifies two individual conditions under which no instances of infighting occurred. The first is the absence of *Imperial past*, and the second is the presence of a *Large population*. These two variables account for 10% and 17%, respectively, of all cases where infighting is absent. While Wimmer and colleagues contend that larger populations would be less likely to experience infighting, our results show that infighting is not merely less likely, but simply does not occur in the states accounted for that have populations exceeding 27 million. Moreover, while Wimmer and colleagues do not present any hypotheses regarding the relationship between an imperial past and the likelihood of infighting, they do argue that infighting will be less likely in less coherent states, and note elsewhere that states with an imperial past are less coherent. Thus, our finding that the absence of any imperial past precludes infighting is consistent with their general line of argumentation, but is not reflected in the statistical analysis.

Regarding the two more complex configurations, we find that (*i*) when at least some portion of the population is systematically excluded from power (**presence** of *Excluded population*), there is no more than one ethnic group in power in the government (**absence** *Center segmentation*), and an ethnolinguistic fractionalization score of 0.2 or lower (**absence** *Linguistic fractionalization*), and (*ii*) when no portion of the population is politically excluded (**absence** of *Excluded population*), the country is classified as high income according to the World Bank (**presence** of *High GDP*), and has an ethnolinguistic fractionalization score of 0.2 or lower (**absence** of *Linguistic fractionalization*), there are no instances of infighting. These configurations account for 37% and 10% of all cases where infighting was absent, respectively. These findings are compelling for several reasons. These configurations illustrate how individual conditions have different effects depending on with which conditions they co-occur (i.e., the effect of *Excluded population*), and demonstrate how sets of conditions—which would be too complex to properly interpret using interaction terms via standard statistical analysis—effectively preclude the outcome of interest. Importantly, these findings complement the findings presented by Wimmer, Cederman, and Min, identifying conditions under which statistically insignificant predictors help us understand the *absence* of the outcome.

This paper offers a novel approach to analyzing rare outcomes. Rather than attempting to fit a very small handful of observations to a standard distribution (i.e., Poisson, Weilbull, negative binomial) in the context of the general linear mode, we provide a complementary approach that focuses on identifying conditions under which the rare event does not occur. Because our approach is configurational, it is not directly comparable to these other methods. Thus, returning to our haystack analogy, our approach can be applied to substantially reduce the size of the haystack, at which point more common regression-based approaches can also be applied.

## Supporting information

S1 Table Truth table for configurational analysis of wimmer, cederman and min (2009). Rows are sorted in descending order by Consistency and N. 47 (un-minimized) configurations representing 3,368 observations (65.32% of observations) are entirely consistent (consistency = 1.000) with the absence of infighting. *Outcome* was coded true (*Outcome =* 1) only for configurations that met the threshold for 100% consistency. Lower consistency scores indicate that at least one observation accounted for in that configuration is an instance of infighting (the rare outcome).
(DOCX)

S1 Appendix R code for the Monte Carlo experiment. (DOCX)
