# Supplementary material for: Precluding rare outcomes by predicting their absence
Source: PLoS One. 2019 Oct 10;14(10):e0223239. doi: 10.1371/journal.pone.0223239 (PMC6786560; doi:10.1371/journal.pone.0223239)
Supplement: S1 Table — Rows are sorted in descending order by Consistency and N. 47 (un-minimized) configurations representing 3,368 observations (65.32% of observations) are entirely consistent (consistency = 1.000) with the absence of infighting. Outcome was coded true (Outcome = 1) only for configurations that met the threshold for 100% consistency. Lower consistency scores indicate that at least one observation accounted for in that configuration is an instance of infighting (the rare outcome). (DOCX) [file pone.0223239.s001.docx]

**Table S1. Truth Table for Configurational Analysis of Wimmer, Cederman and Min (2009)**

| Excluded Population | Center Segmentation | Imperial Past | Linguistic Fractionalization | High GDP | Large Population | Ongoing War | N (Cases) | Outcome | Consistency | |
| --- | --- | --- | --- | --- | --- | --- | --- | --- | --- | --- |
| 1 | 0 | 1 | 0 | 0 | 0 | 0 | 900 | 1 | 1 |  |
| 0 | 0 | 1 | 0 | 1 | 0 | 0 | 378 | 1 | 1 |  |
| 1 | 0 | 1 | 0 | 1 | 0 | 0 | 342 | 1 | 1 |  |
| 1 | 0 | 1 | 0 | 0 | 1 | 0 | 258 | 1 | 1 |  |
| 1 | 1 | 1 | 1 | 0 | 0 | 0 | 190 | 1 | 1 |  |
| 1 | 0 | 0 | 1 | 0 | 0 | 0 | 136 | 1 | 1 |  |
| 1 | 0 | 0 | 0 | 1 | 1 | 0 | 135 | 1 | 1 |  |
| 1 | 0 | 1 | 0 | 1 | 1 | 0 | 107 | 1 | 1 |  |
| 1 | 0 | 1 | 1 | 1 | 0 | 0 | 104 | 1 | 1 |  |
| 1 | 0 | 1 | 1 | 0 | 1 | 0 | 82 | 1 | 1 |  |
| 1 | 1 | 1 | 1 | 0 | 1 | 0 | 81 | 1 | 1 |  |
| 0 | 0 | 1 | 0 | 0 | 1 | 0 | 71 | 1 | 1 |  |
| 0 | 1 | 0 | 1 | 1 | 0 | 0 | 55 | 1 | 1 |  |
| 1 | 0 | 0 | 0 | 0 | 0 | 0 | 54 | 1 | 1 |  |
| 0 | 0 | 1 | 0 | 1 | 1 | 0 | 53 | 1 | 1 |  |
| 0 | 0 | 0 | 0 | 1 | 0 | 0 | 52 | 1 | 1 |  |
| 1 | 0 | 1 | 0 | 0 | 0 | 1 | 50 | 1 | 1 |  |
| 1 | 0 | 1 | 1 | 0 | 0 | 1 | 48 | 1 | 1 |  |
| 1 | 0 | 0 | 0 | 0 | 1 | 0 | 36 | 1 | 1 |  |
| 0 | 1 | 1 | 1 | 1 | 0 | 0 | 29 | 1 | 1 |  |
| 0 | 0 | 0 | 0 | 0 | 0 | 0 | 27 | 1 | 1 |  |
| 1 | 0 | 1 | 1 | 0 | 1 | 1 | 27 | 1 | 1 |  |
| 0 | 0 | 1 | 0 | 0 | 0 | 1 | 20 | 1 | 1 |  |
| 1 | 0 | 1 | 1 | 1 | 1 | 0 | 18 | 1 | 1 |  |
| 1 | 1 | 0 | 1 | 0 | 0 | 0 | 17 | 1 | 1 |  |
| **Truth Table, Cont.** | | |  |  |  |  |  |  |  |  |
| Excluded Population | Center Segmentation | Imperial Past | Linguistic Fractionalization | High GDP | Large Population | Ongoing War | N (Cases) | Outcome | Consistency |  |
| 1 | 1 | 1 | 1 | 0 | 1 | 1 | 13 | 1 | 1 |  |
| 0 | 1 | 1 | 0 | 1 | 0 | 0 | 11 | 1 | 1 |  |
| 1 | 0 | 0 | 0 | 0 | 0 | 1 | 11 | 1 | 1 |  |
| 1 | 1 | 1 | 0 | 1 | 1 | 0 | 9 | 1 | 1 |  |
| 1 | 0 | 1 | 0 | 0 | 1 | 1 | 9 | 1 | 1 |  |
| 0 | 0 | 1 | 1 | 0 | 1 | 0 | 8 | 1 | 1 |  |
| 1 | 0 | 0 | 1 | 1 | 1 | 0 | 8 | 1 | 1 |  |
| 0 | 1 | 1 | 1 | 0 | 1 | 0 | 5 | 1 | 1 |  |
| 1 | 1 | 1 | 1 | 1 | 0 | 0 | 3 | 1 | 1 |  |
| 1 | 0 | 0 | 1 | 0 | 1 | 0 | 3 | 1 | 1 |  |
| 1 | 1 | 1 | 0 | 0 | 0 | 1 | 3 | 1 | 1 |  |
| 1 | 1 | 0 | 1 | 0 | 0 | 1 | 3 | 1 | 1 |  |
| 0 | 0 | 1 | 0 | 0 | 1 | 1 | 2 | 1 | 1 |  |
| 0 | 1 | 1 | 1 | 0 | 1 | 1 | 2 | 1 | 1 |  |
| 0 | 0 | 0 | 0 | 0 | 0 | 1 | 1 | 1 | 1 |  |
| 0 | 1 | 1 | 0 | 0 | 0 | 1 | 1 | 1 | 1 |  |
| 1 | 0 | 0 | 1 | 0 | 0 | 1 | 1 | 1 | 1 |  |
| 0 | 0 | 1 | 1 | 0 | 0 | 1 | 1 | 1 | 1 |  |
| 0 | 0 | 0 | 0 | 1 | 0 | 1 | 1 | 1 | 1 |  |
| 1 | 0 | 1 | 0 | 1 | 0 | 1 | 1 | 1 | 1 |  |
| 1 | 0 | 0 | 1 | 0 | 1 | 1 | 1 | 1 | 1 |  |
| 1 | 1 | 1 | 0 | 1 | 1 | 1 | 1 | 1 | 1 |  |
| 0 | 1 | 1 | 1 | 0 | 0 | 0 | 304 | 0 | 0.996711 |  |
| 1 | 0 | 1 | 1 | 0 | 0 | 0 | 594 | 0 | 0.996633 |  |
| 0 | 0 | 1 | 0 | 0 | 0 | 0 | 616 | 0 | 0.99513 |  |
| 1 | 1 | 1 | 0 | 0 | 0 | 0 | 67 | 0 | 0.985075 |  |
| **Truth Table, Cont.** | | |  |  |  |  |  |  |  |  |
| Excluded Population | Center Segmentation | Imperial Past | Linguistic Fractionalization | High GDP | Large Population | Ongoing War | N (Cases) | Outcome | Consistency |  |
| 0 | 0 | 1 | 1 | 0 | 0 | 0 | 73 | 0 | 0.972603 |  |
| 1 | 1 | 1 | 0 | 1 | 0 | 0 | 32 | 0 | 0.96875 |  |
| 0 | 0 | 1 | 1 | 1 | 0 | 0 | 20 | 0 | 0.95 |  |
| 1 | 1 | 1 | 1 | 0 | 0 | 1 | 15 | 0 | 0.933333 |  |
| 0 | 1 | 1 | 1 | 0 | 0 | 1 | 3 | 0 | 0.666667 |  |
