# Supplementary material for: Precluding rare outcomes by predicting their absence
Source: PLoS One. 2019 Oct 10;14(10):e0223239. doi: 10.1371/journal.pone.0223239 (PMC6786560; doi:10.1371/journal.pone.0223239)
Supplement: S1 Appendix — (DOCX) [file pone.0223239.s002.docx]

**R Code for the Monte Carlo Experiment**

# Will need the QCA package for the TruthTables

#install.packages(QCA)

# load the package

require(QCA)

# Generate a function that simulates data

# The function takes a number of oberservations, the number of

# predictors, and the proportion of 1's in the outcome, # respectively

simdat <- function(nobs = 500, p = 20,prop.ones=.04){

X <-matrix(rnorm(nobs *(p-1)), ncol=(p-1))

# For predictors 1:19, the probability of a 1 is .159 and is # random

X[,1:(p-1)] <- (X[,1:(p-1)] > 1) * 1

# The outcome is a series of 1s followed by 0s

y<-rep(0,nobs)

num.ones<-prop.ones*nobs

y[1:num.ones]<-1

# Defining the "key" predictor

first<-rep(0,nobs)

# for the first num.ones cases, make the probability of a 1 to # be .3

first[1:num.ones]<-.3

# for the rest of the cases, make the probability of a 1 small

first[num.ones+1:nobs]<-num.ones/2/(nobs-num.ones)

# turn the probabilities into zeroes and ones

second<-rbinom(nobs,1,first)

# generate the output

out<-as.matrix(cbind(y,X,second))

return(out)}

# Design space of the monte carlo experiment

nobs.sim<-c(rep(500,1000),rep(1000,1000),rep(2000,1000),rep(4000,1000),rep(8000,1000))

prop.ones.sim<-c(rep(.04,5000),rep(.02,5000),rep(.01,5000),rep(.005,5000),rep(.0025,5000))

nobs.sim<-rep(nobs.sim,5)

# define vectors that will be replaced in the loop

prop.removed<-rep(0,25000)

# This loop pulls out the sample size and proportion of ones, # simulates data, and then uses #the Truth Table to determine # the cases that can be removed

for(i in 1:25000){

nobs<-nobs.sim[i]

prop.ones<-prop.ones.sim[i]

X<-simdat(nobs=nobs,prop.ones=prop.ones)

y<-X[,1]

X<-X[,2:21]

simconfig3<-data.frame(y,X)

tt<-truthTable(simconfig3, outcome="y", neg.out=TRUE, conditions=(colnames(simconfig3)[-1]), n.cut=2, incl.cut=1, sort.by=c("incl", "n"), decreasing=TRUE)$tt

# identify which rows of the truth table include cases that can be removed

theserows<-which(tt[,which(colnames(tt)=="OUT")]==1)

prop.removed[i]<-(sum(tt[theserows,which(colnames(tt)=="n")]))/nobs}

# End the loop

results.1<-cbind(prop.removed,nobs.sim,prop.ones.sim)
